# Curiosity in a Novel Virtual Reality Scenario and Its Association With Symptoms of Depression: Observational Pilot Investigation

**DOI:** 10.2196/80120

**Published:** 2026-05-04

**Authors:** Emma Therése Eliasson, Sara Sutori, Francesca Mura, Victor Ortiz, Vincenzo Catrambone, Gergö Hadlaczky, Ivo Todorov, Antonio Luca Alfeo, Valentina Cardi, Mario G C A Cimino, Giovanna Mioni, Mariano Alcañiz Raya, Gaetano Valenza, Vladimir Carli, Claudio Gentili

**Affiliations:** 1 National Centre for Suicide Research and Prevention (NASP), Department of Learning, Informatics, Management and Ethics (LIME), Karolinska Institutet, Granits väg 4, Solna, 17177, Sweden, 46 701407295; 2 Padova Neuroscience Center (PNC), University of Padua, Padua, Italy; 3 Instituto Universitario de Investigación en Tecnología Centrada en el Ser Humano, Universitat Politècnica de València, Ciudad Politécnica de la Innovación, València, Spain; 4 Research Center E. Piaggio, Department of Information Engineering, University of Pisa, Pisa, Italy; 5 Department of Theoretical and Applied Sciences, Università degli Studi eCampus, Novedrate, Italy; 6 Department of General Psychology, University of Padua, Padua, Italy

**Keywords:** virtual reality, curiosity, depressive symptoms, symptom assessment, exploratory behavior

## Abstract

**Background:**

Curiosity plays a fundamental role in human learning, development, and motivation, and emerging evidence suggests that reduced curiosity is linked to poorer mental health outcomes, including depressive symptoms (DS). However, to date, the majority of curiosity research relies on self-report assessments and thus risks biased reporting. Virtual reality (VR), a novel tool increasingly used within mental health research and treatment, might represent a potent tool for offering ecologically valid insights into curiosity-driven behaviors while circumventing issues related to self-report assessments, including demand characteristics and recall bias.

**Objective:**

The study aimed to enhance the assessment of curiosity by using a novel VR environment and to examine its relevance to DS. Specifically, we tested 2 hypotheses using a novel VR environment: first, that curiosity, as assessed through spontaneous exploratory interactions and behaviors in VR, positively correlates with self-reported curiosity, and second, that VR-based curiosity is inversely associated with DS.

**Methods:**

This exploratory study used an observational design that included 100 volunteers. All participants completed self-reported assessments of DS and curiosity before engaging in a novel VR scenario. Although progression in the virtual environment required solving cognitive tasks, these were embedded as structural elements rather than framed as the primary objective. Instead, participants’ free explorations and interactions with objects formed the basis for the 4 curiosity metrics used in this study. After VR exposure, participants completed a questionnaire assessing cybersickness symptoms.

**Results:**

Hypothesis 1 was not supported, as only one curiosity metric, namely object interactions, was positively associated with one aspect of curiosity relating to motivation to seek new knowledge and experiences. Further, diminishing significance after correction for multiple testing warranted caution. Results relating to hypothesis 2 indicated partial support, in that object interaction was significantly associated with DS while controlling for age, sex, and cybersickness levels. Sensitivity analyses showed no associations between object interactions and self-reported anxiety and stress symptoms.

**Conclusions:**

VR may be a potent tool for assessing exploratory behaviors in a controlled, yet ecologically valid, environment that avoids issues related to self-report. However, whether such motivations translate to established curiosity constructs warrants further research. This study also provided preliminary insights into how assessing exploratory interactions in VR may be a promising avenue that could enhance the understanding of the etiology and assessment of DS—particularly its early stages.

## Introduction

### Background

The human inclination for curiosity, typically characterized as the propensity to seek out novel, complex, and challenging situations [1,2], is deeply woven into the fabric of our everyday experiences—oftentimes to the extent that we “[a]re nearly oblivious to its pervasiveness in our lives” ([3], p. 449). Curiosity is multifaceted, in that it can vary momentarily [4], as well as being measured as a somewhat stable trait [5]. Being a key ingredient to human learning and development [3,6], it is an essential component of intrinsic motivation and a core mechanism of the reward sensitivity system [1,3]. As encouragement to engage with novel situations not only fosters learning and development, but also accrual of resources, higher curiosity has been associated with a range of positive outcomes, including increased psychological, emotional, and social well-being [7], reduced stress-related negative affect [8], and better social and emotional skills [9]. Given its importance, encouraging aspects of curiosity have been the target of several interventions (for a meta-analysis, see [10]), including enhancing curiosity through manipulating the timing of new information [11], school interventions aimed at fostering investigative interests and desire to obtain new knowledge [12], and through virtual environments encouraging exploration [13].

Lower curiosity has been associated with adverse mental health symptoms, with depression in particular consistently linked to a reduced tendency for curiosity [14,15]. This association is perhaps not unexpected given the etiology of depression, where one of the core symptoms, in addition to persistent low mood, is lack of motivation and loss of interest in activities [16]. Motivation is thus seen as a key factor to understanding depression, not only regarding contributing to its onset but also as a crucial mechanism underpinning symptom relief following successful treatment regimens [17,18]. Several longitudinal studies have also given insight into the link between curiosity and affective symptoms, shedding further light on their intertwined relationship. For instance, in a 1-year follow-up study, Sheldon et al [19] found that higher baseline trait curiosity together with high goal attainment were linked to better well-being levels at follow-up. Moreover, using a 21-day daily diary study design, Lydon‐Staley et al [4] demonstrated that lower curiosity lability (ie, the extent to which curiosity fluctuated across days) was associated with higher life satisfaction and lower depressed mood, highlighting a role of momentary within‐person variations in curiosity and the propensity for depressive symptoms (DS). In a subsequent analysis of the same study sample, Drake et al [8] found that stressor-related negative mood was lower on days when curiosity was higher, indicating potential resilience-related benefits of curiosity when facing stress. One recent study further highlighted a potentially protective role of curiosity, where adults with higher self-reported curiosity during adolescence reported lower DS in adulthood [20], even though the cross-sectional design, and thus risk of recall bias, should be noted.

The most common way in which curiosity has been assessed is through self-report measures [21,22], where the construct has been conceptualized in a variety of ways, using both unidimensional [23] and multidimensional scales [24], with the latter being more commonly used [22]. For instance, a commonly used measure, namely the Curiosity and Exploration Inventory-II [5], assesses curiosity along 2 dimensions: stretching (referring to the motivation to seek out new knowledge and experiences) and embracing (the willingness to tolerate and engage with uncertainty and novelty in a positive manner). More recently, Kashdan et al [14] developed a 5-dimensional model of curiosity, namely the 5-Dimensional Curiosity Scale, encompassing joyous exploration, deprivation sensitivity, stress tolerance, social curiosity, and thrill seeking.

However, while self-report measures are widely used [22], inherent drawbacks exist in assessing curiosity through such scales, including the risk of demand characteristics [25] and recall bias [26]. In other words, it is not unlikely that the desire to describe oneself as curious and motivated distorts a person’s true propensity to act as such in their everyday lives. On the contrary, while self-report curiosity scales have given valuable insights into its associations with mood symptoms (eg, [4,8,19]), a negative emotional recall bias and diminished retrieval of positive memories, theorized as a core cognitive characteristic of depression [26], may lead individuals with DS to underestimate their propensity for curiosity in their everyday lives—potentially distorting the true relationship between DS and curiosity. Likewise, behavioral aspects of curiosity and motivation, including approach toward novel stimuli, active information sampling, and environmental engagement [3,27], may be difficult to accurately capture via self-report. Translational paradigms adapted from rodent open-field and hole-board tasks have started to operationalize these behavioral expressions in humans, primarily as spatial exploration, which has also shown sensitivity to psychiatric symptom dimensions [28-30]. Although contemporary theories characterize curiosity as a multifaceted construct encompassing epistemic, perceptual, and intrinsic dimensions [2,3], many accounts converge in identifying information-seeking and exploratory behavior as key behavioral expressions of this construct [31]. Within this framework, exploratory actions may represent observable expressions of curiosity-related processes, even if the curiosity construct in its entirety is broader [2,3]. Immersive virtual reality (VR) paradigms could offer a controlled yet ecologically valid context to quantify such outputs. Accordingly, exploratory behaviors could be depicted, not as synonymous with curiosity in its entirety but as a theoretically grounded behavioral proxy reflecting underlying exploratory motivation.

Issues inherent in self-report assessments have also been highlighted as a general obstacle to developing effective and reliable diagnostic tools for depression [32]. This is particularly relevant given that such tools are not only vulnerable to symptom insight and subjective interpretation of questionnaires but can also be influenced by variability in how clinicians present questions and response alternatives [26,33,34]. Given these issues, several attempts to move beyond subjective assessment methods by using technological advancements, such as speech-analysis [35], gamification [36], or VR [37-39] have been made. Such tools can be particularly useful in providing more sophisticated, yet structured frameworks to evaluate early and often subtle symptoms that may otherwise go unnoticed, thus enabling early detection and prevention. VR, in particular, has been suggested as an important avenue to support improvements regarding both treatments of mental health conditions [40], as well as diagnostic accuracy and early symptom detection [37,41]. Reviews synthesizing VR applications across psychiatric disorders indicate that the field has predominantly focused on anxiety-related conditions in the context of exposure-based paradigms, with depression remaining comparatively undertargeted within the broader landscape of VR-based mental health research [42-44]. While still comparatively limited in scope, the growing body of research at the intersection of VR applications and depression has resulted in several dedicated reviews [45-47]. However, broad reviews [43], as well as depression-specific syntheses, consistently indicate that most existing studies focus on treatment-oriented applications, including symptom reduction, behavioral activation, or emotion regulation—rather than on the assessment of depressive symptomatology (eg, see [37,40,48]).

To date, only a few studies have used VR to aid depression symptom assessment [39,49,50]. Illustrating its potential to improve diagnostic precision, Voinescu et al [50] used a novel VR task to test attentional abilities among individuals with low and elevated symptoms of anxiety and depression. They found that participants’ task performance significantly correlated with symptoms of depression and anxiety above traditional cognitive measures. Duan and colleagues [49] used a novel VR environment to assess self-blame related tendencies and found that feeling like punishing oneself predicted poorer depression prognosis over 4 subsequent months of primary care treatment. Moreover, our research group recently incorporated a range of VR-based metrics, including eye-movements, cognitive tasks, physiological indicators, as well as curiosity in the form of exploration behaviors, to classify the presence or absence of DS, demonstrating the utility of using multimodal measurements in supporting VR-based diagnostics [39].

However, to our knowledge, no prior VR study has isolated and examined exploratory tendencies as behavioral indicators of curiosity or investigated their association with DS, including their specificity relative to related constructs, namely anxiety and stress. Given that subtle alterations in motivational tendencies can serve as both early signs of depression and potential risk factors for poorer treatment outcome [49,51], VR might represent a potent tool for offering insights into curiosity-driven behaviors, while circumventing issues of demand characteristics and depressive recall distortions. Such advancements could not only help further research on the early detection of at-risk individuals and enhance the specificity of predicting DS but could also support the development of personalized VR-based therapies with the potential to adapt to individual motivational profiles.

### Aim and Hypotheses

The aim of this pilot investigation was to test the possibility of assessing curiosity through spontaneous interactions within a novel VR environment, and whether curiosity assessed this way is associated with severity of DS. More specifically, the following 2 exploratory hypotheses guided this study:

1. Curiosity as assessed through spontaneous interactions within a novel VR environment will be positively associated with curiosity as assessed by the Curiosity and Exploration Inventory-II (CEI-II [5]).
2. Curiosity as assessed through spontaneous interactions within a novel VR environment will be inversely associated with DS as assessed by the depression subscale of the Depression Anxiety and Stress Scales-21 items (DASS-21 [52]).

## Methods

### Design

This was an exploratory study with an observational design. Data collection was conducted at the University of Padua, Italy. All participants underwent a VR engagement in the form of a VR serious game, described in more detail below. The study described in this paper represents a secondary analysis conducted as part of our larger project with the aim to investigate the potential of integrating VR and explainable artificial intelligence to differentiate between healthy volunteers and individuals with DS, with the overall aim to support the enhancement of depression screening (see [39] and [38]).

### Participant Recruitment

Enrollment in the study occurred between November 2022 and November 2023. Potential participants were recruited through flyers at the University of Padua or by having taken part in other studies conducted by the same research group. Interested participants were directed to an online platform providing more information about the study, an online consent form, and a survey (administered in Qualtrics) to screen inclusion and exclusion criteria (detailed later).

### Inclusion Criteria

To be included in the study, participants had to be aged between 18 and 35 years and be free from any condition that could impair the ability to interact with the VR environment (such as visual impairment without the possibility of correction via lenses). Moreover, given the aim of the larger study, which was to investigate the potential of integrating VR and explainable artificial intelligence to differentiate between healthy volunteers and individuals with DS based on the Patient Health Questionnaire-9 (PHQ-9, [53]), the screening procedure included a PHQ-9 assessment (see [39]). More specifically, participants were included if they scored 9 or above (allocated to the DS group) or 5 or below (allocated to the healthy control [HC] group). Additional inclusion criteria for the DS group included the lack of a diagnosis of any other psychiatric disorders than depressive or anxiety disorders and treatment stability over the last 4 weeks if they received psychological or pharmacological treatment at the time of assessment. Participants in the control group had to be free from any previous or current psychiatric disorders. Recruitment was completed when the predefined target sample size of 100 was reached (n=50 participants in the HC group and n=50 participants in the DS group). However, while the original study [39] divided participants into two groups based on their PHQ-9 score as described above, this division was not relevant to this study as the DASS-21 [52] was used as a continuous outcome variable for the full study sample (see further details in the description of measures below).

### Preregistration

The project on which this exploratory study is based was preregistered on the ISRCTN registry [54]. After protocol registration, 1 modification to the study protocol was made, concerning the threshold for inclusion into the study based on current depressive symptom severity using the PHQ-9 [53]. Due to the difficulty in recruiting volunteers in line with the main study’s timeline, the upper threshold for inclusion in the control group was increased from 4 to 5, while the lower threshold to be included in the group with DS was decreased from 10 to 9.

### Ethical Considerations

The study was carried out in accordance with the Declaration of Helsinki and obtained ethical approval from the local ethical committees’ at the sites where data were collected (Italy) and analyzed (Italy and Sweden). Approval was granted in Italy by Comitato Etico della Ricerca Psicologica (Area 17), protocol number 4688, and in Sweden, by the Etikprövningsmyndigheten (2023-00959-01). Written informed consent was obtained from all participants prior to study participation, where emphasis was placed on the voluntary nature of participation and the possibility to withdraw from the study at any time, without reason, or any penalty. All participants were informed about data privacy and confidentiality. All information collected for this research project was handled in compliance with D.Lgs.196/2003, EU GDPR 679/2016, and Article 9 of the Code of Ethics of Italian Psychologists. Participant data were pseudonymized upon collection through the assignment of unique identification codes. The key linking these codes to personal identifiers was stored separately by the data manager (University of Padua) and restricted to authorized personnel only. All analyses were conducted on pseudonymized datasets. No directly identifiable participant information is included in this manuscript or supplementary materials. All participants provided informed consent for the collection, use, and publication of their data for research purposes. Study compensation included €25 (worth between US $24.38 and US $28.14 over the duration of the recruitment period).

### Technological Specifications

#### System Overview

The VR system was built around the HP Reverb G2 Omnicept Edition headset, which features multiple biometric sensors, including eye tracking, a lower facial camera, a heart rate monitor, and sensors for brain activity. It retains the core specifications of the base headset and has a resolution of 2160×2160 pixels per eye, a 114° field of view, a 90 Hz refresh rate, and integrated audio. The virtual environment was developed in Unity 2020.3.39 LTS, using the HP Omnicept SDK and OpenXR to maximize compatibility and platform versatility. Development processes, including occlusion culling, foveated rendering, asset optimization, and baked lighting, were used to ensure a smooth and effective environment.

#### Virtual Environment

**Figure 1.**
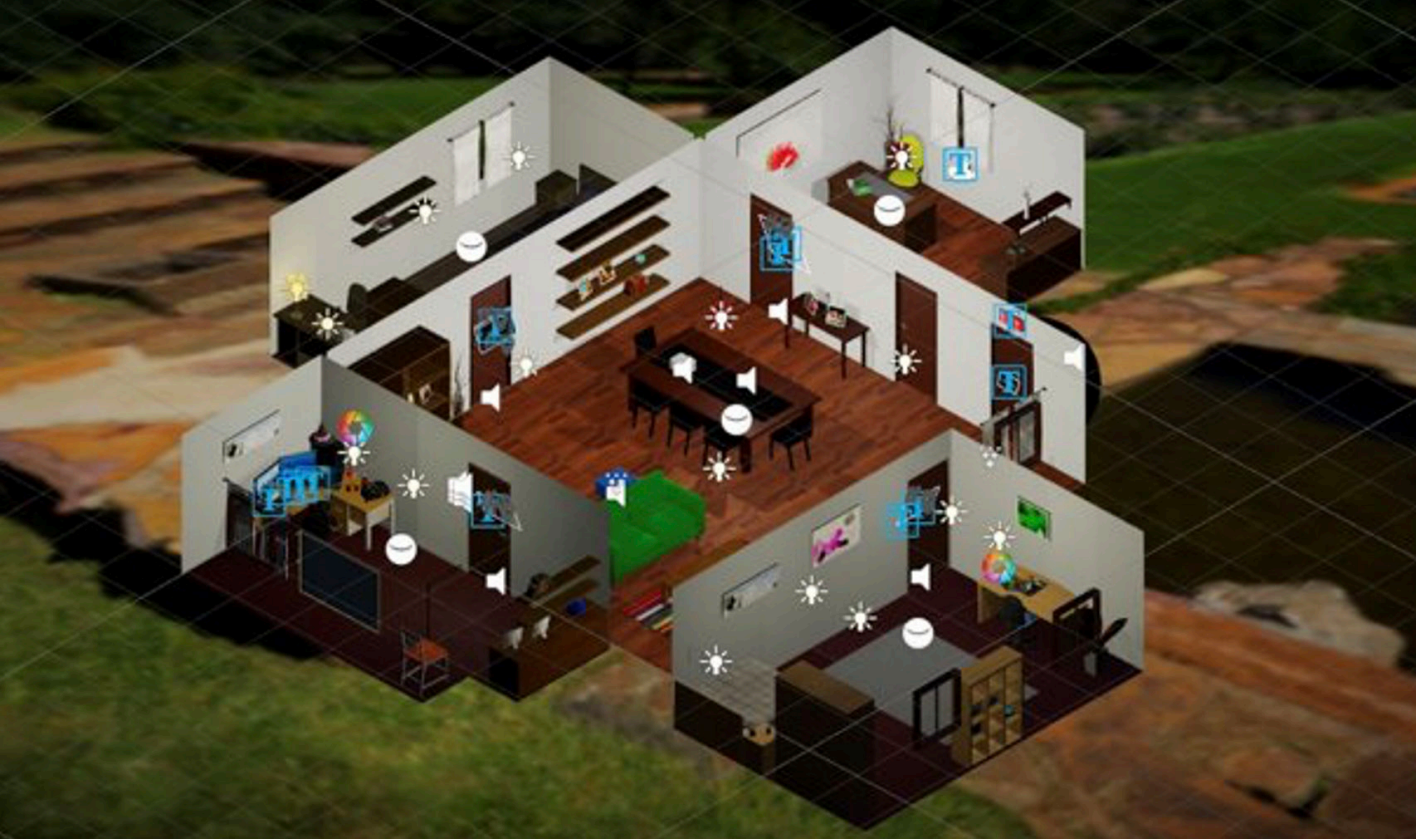
Overhead view of the interactive virtual reality environment, resembling a family home. Participants freely explored interconnected rooms and unlocked doors by completing embedded cognitive tasks.

The VR environment was originally developed to differentiate between HCs and individuals with DS, based on cognitive, behavioral, and physiological data [39]. It was therefore designed to evoke certain behaviors and responses hypothesized to be associated with depression, including cognition (attention, working memory, processing speed, executive functioning, and cognitive flexibility), metacognition, persistence or grit, behavioral and attentional biases toward negative stimuli, speed, as well as curiosity—the latter of which was of interest for the current study. During the VR interaction, physiological data were recorded, including skin conductance, heart rate variability, and eye-tracking. More details of these results, and of the overall performance of the environment in its entirety, including acceptability, are discussed elsewhere [38,39]. Figure 1 shows a depiction of the environment, which was built to resemble a multiroom family home consisting of 4 rooms, each with different lighting parameters. Within each room, various stimuli and interactive elements were present, such as sound boxes, a chalkboard, and other objects that could be interacted with, including items that could be picked up and thrown, musical buttons, and photos that could be picked up and inspected. In addition, another room could be visually explored via a keyhole in a door. Every interaction participant made in the environment was recorded. For the purpose of the current study, data on interactions with environment objects, data on key-hole interactions, as well as overall time spent exploring the environment were extracted (see *Measures* section for a further explanation on the chosen curiosity indices).

#### VR Scenario

VR engagement started with a tutorial showing participants the controllers and how to move around in the environment and interact with objects. The tutorial detailed that in order to finish the VR scenario, they had to complete 4 cognitive tasks (Rapid Visual Information Processing; N-back (2-back); Trail Making Test (parts A and B); and Wisconsin Card Sorting Test). Completion of each task led to the opening of a door that enabled participants to move freely between the rooms and explore aspects of each respective room if they wished to. For more information on specific tutorial instructions, see tutorial script in Supplementary Materials. A study administrator used an interface to manage the experiment and observe participant actions in real time. The interface included control options to maintain smooth session flow, such as terminating a cognitive task upon participant request. An additional camera mounted on the participant enabled effective monitoring of overall progress.

### Measures

Although only outcomes relevant to this study are outlined below, a full list of outcomes used for the larger study is detailed in earlier publications [38,39].

#### Participant Characteristics

All participants filled in a background questionnaire detailing demographic characteristics and other background information including medication use. For the purpose of the current study, we report on sex, age, education, and antidepressant medication use.

#### Depression Anxiety and Stress Scales

The DASS-21 [52] is a 21-item scale used to assess state symptoms of depression (DASS-Depression [DASS-D], 7 items), anxiety (DASS-Anxiety [DASS-A], 7 items), and stress (DASS-Stress [DASS-S], 7 items), where the severity of each symptom is scored from 0 “Did not apply to me at all” to 3 “Applied to me very much or most of the time.” The Italian version of the DASS-21 and its respective subscales have been validated in both clinical and nonclinical populations and shown to have good internal consistency in both clinical and nonclinical populations (DASS-D: *α*=.91, .82; DASS-A: *α*=.88, .74; DASS-S: *α*=.83, .84; DASS-21: *α*=.92, .90), as well as good convergent and divergent validity [55]. Given that the DASS-21 was developed as a dimensional symptom measure, rather than a diagnostic categorical tool [52,56], we based our statistical analyses on continuous scores, thereby also avoiding information loss and reduced power associated with categorizing continuous variables [57]. While the main study (see [38,39,54]), on which this exploratory study is based, used the PHQ-9 as the basis for the main categorical outcome (DS vs HC), the DASS-21 was deemed more appropriate as the main outcome for the purpose of the current study. While the scales are highly correlated (eg, [58,59]), the DASS-D scale was chosen due to its narrower focus on emotional and cognitive aspects of DS [60] that may be more relevant to nonclinical populations. On the contrary, as the PHQ-9 criteria are designed to match the DSM (*Diagnostic and Statistical Manual of Mental Disorder*s) criteria for depression [53], this is more appropriate when the presence of DS is a categorical outcome [38,39]. Moreover, while the DASS-D served as the main measure of DS, sensitivity analyses were conducted using the DASS-A and DASS-S subscales to examine the specificity of the association between VR curiosity and DS.

#### Curiosity and Exploration Inventory-II

The CEI-II [5] was used to assess self-reported curiosity. The inventory consists of 10 curiosity items, containing 2 subscales, namely embracing and stretching. Embracing (5 items) refers to the willingness to embrace the uncertain, novel, and unpredictable nature of everyday life, whereas the stretching subscale (5 items) assesses motivation to seek new knowledge and experiences. Items are scored on a scale of 1 “very slightly or not at all” to 5 “extremely.” The CEI-II is a widely used tool to assess curiosity and has shown good to acceptable internal consistency (CEI-II total *α*=.86‐.83, CEI-II stretching *α*=.79‐.80, and CEI-II embracing *α*=.75‐.79) [5]. For the purpose of this study, the scale was translated into Italian. The initial translation was carried out by a native speaker. Subsequently, two independent native speakers reviewed the Italian version to check the accuracy and quality of the translation. Internal consistency for the Italian version used in this study was CEI-II total *α*=.83, CEI-II stretching *α*=.79, and CEI-II embracing *α*=.75.

#### Simulator Sickness Questionnaire

The Simulator Sickness Questionnaire (SSQ) [61] was used to assess levels of cybersickness following VR exposure. The questionnaire contains 16 symptoms, which are rated on a scale of subjective severity: 0 (none), 1 (slight), 2 (moderate), and 3 (severe). In addition to obtaining a total score, separate scores for nausea, oculomotor, and disorientation can be obtained [61]. For the purpose of this study, the SSQ total score was added as a covariate, in order to control for potential cybersickness that might affect propensity to explore VR environments. Internal consistency for the Italian version used in this study was *α*=.82.

#### VR-Based Curiosity

Curiosity in VR was assessed based on several prespecified behaviors, surrounding participants’ propensity to interact with various VR features, including the variety of objects interacted with, time spent interacting with objects, number of objects interacted with, time spent looking through a keyhole into a room with ambiguous conversational voices, overall time spent exploring the environment (excluding required cognitive tasks), and laser gun-related interactions. These behaviors were conceptualized as observable expressions of exploratory engagement. While not direct measures of curiosity as a motivational construct, such approach-oriented actions may reflect behavioral correlates of information-seeking and novelty engagement described in theoretical models of curiosity [2,3].

The selection of these indices was informed by reverse-translational paradigms in which discrete object interactions index exploratory behavior [28]. Accordingly, object-related interactions formed the primary behavioral anchor of this study. Given the high intercorrelations among the object-related variables (variety of objects, number of objects, and time spent interacting with objects; Figure S1 in Multimedia Appendix 1), these indices were combined into a single composite–object interactions–using rank-based methods to accommodate nonnormal distributions. The composite was created primarily to address statistical redundancy and reduce multicollinearity in subsequent analyses. Behaviorally, this index can be interpreted as a summary measure of overall object-directed exploration, capturing both the breadth (number and variety of objects) and depth (time spent) of interaction with objects. Importantly, this composite represents a pragmatic operationalization of object interaction-based exploration rather than a theoretically predefined latent construct. Conceptually, object interactions can be considered most closely aligned with approach-oriented dimensions of curiosity, such as CEI-II “stretching” and “joyous exploration” [5,62].

The keyhole metric was intended to map to ambiguity-driven information seeking (information-gap theory [2]). We also analyzed laser gun–related interactions separately as we reasoned that shooting behavior may reflect a distinct form of curiosity that involves both active manipulation and observable consequences in the environment and may be considered as more adjacent to novelty- or thrill-related facets, more closely aligned with the “embracing” aspect of curiosity [5,14]. Time exploring was retained as a general indicator of sustained environmental engagement without assuming direct correspondence to a specific questionnaire dimension.

During preprocessing, shooting with the gun was binary coded (whether participants have shot at least once) due to skewness and zero inflation. Data regarding the keyhole variable was excluded for 1 participant due to a likely data capture error significantly overestimating the time spent looking through the keyhole.

Although theoretically motivated, the absence of prior empirical work operationalizing curiosity behaviorally in immersive VR contexts necessitated an exploratory component. The present approach should therefore be understood as hypothesis-guided but exploratory in its operationalization.

The final VR curiosity variables included in the analysis thus consisted of the following:

- Object interactions (combining time, number, and variety)
- Time spent exploring (time spent exploring the environment, excluding the required cognitive tasks)
- Keyhole (time spent looking through the keyhole)
- Gunshot binary (whether participants shot with the gun or not)

### Study Procedure

Participants who met the inclusion criteria via the online screening tool were invited into the testing lab at the University of Padua, whereby those who wished to proceed provided written informed consent and completed a pre-VR questionnaire battery consisting of providing demographic information and completing the DASS-21 [52] and the CEI-II [5]. Following this, the VR engagement began. After calibrating the headset and eye-tracking, the VR scenario commenced, which started with the tutorial. Following tutorial completion, participants were free to explore the environment and start the cognitive tasks. While the tutorial explained that the environment could be explored, where participants were encouraged to look around, participants were not instructed that their exploratory and interactive behavior was part of the outcome, in order to avoid demand characteristics and to keep the assessment of curiosity as ecologically valid as possible. Following the VR engagement, participants completed the SSQ to assess cybersickness symptoms.

### Analysis

Spearman correlations were used to test the first hypothesis, due to the nonnormalcy of the variables. To test whether VR curiosity variables are associated with depression symptoms while controlling for sex, age, and cybersickness (hypothesis 2), forward stepwise regression analyses were conducted, where curiosity variables were added, 1 at a time, to a base model consisting of the control variables. Given the skewness and nonnormal distribution of the dependent variable which violates a key assumption of ordinary least squares regression, robust regression using *M*-estimation was conducted [63]. Furthermore, the regular regression models resulted in nonnormal residuals and the presence of 7% influential points as indicated by Cook distance; issues that robust regression is designed to handle [63]. To perform sensitivity analyses, similar models were fitted with DASS-A and DASS-S scales as respective outcomes. All analyses were conducted using R (version 4.2.2). The Modern Applied Statistics with S package [64] was used to perform robust linear regression modeling using *M*-estimation via the *rlm*() function. To obtain robust SEs and *P* values, the *sandwich* [65,66] and *lmtest [67]* packages were used. To address potential inflation of type I error due to multiple testing, false discovery rate (FDR) correction using the Benjamini-Hochberg procedure was applied separately to (1) hypothesis 1: the correlation analyses between self-reported and VR-based curiosity measures (3 self-reported×4 VR-based=12 models) and (2) hypothesis 2: the regression models examining associations between VR-based curiosity and DS (4 VR-based×1 depression outcome=4 models).

## Results

### Overview

**Figure 2.**
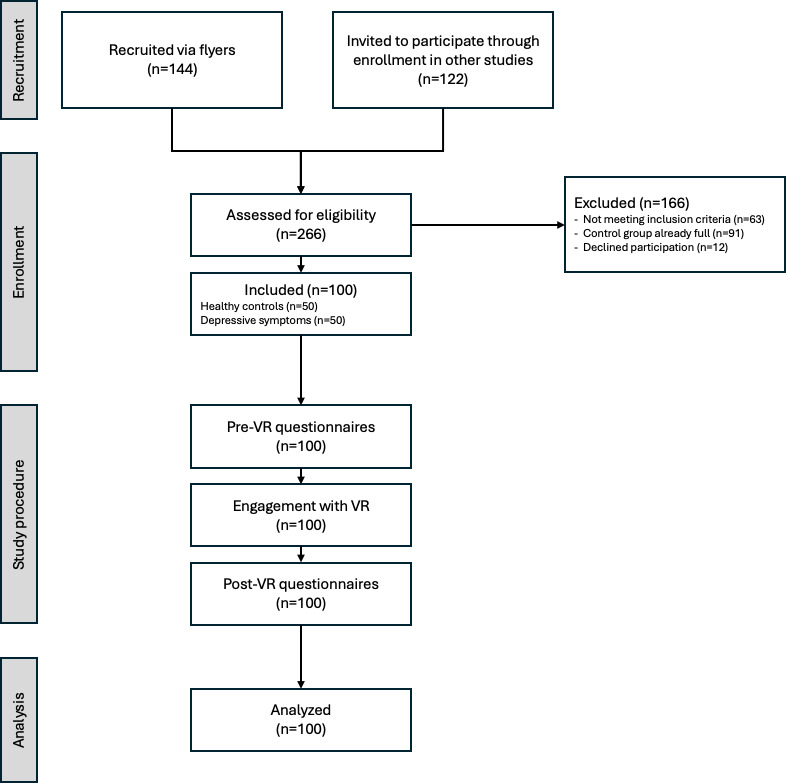
STROBE (Strengthening the Reporting of Observational Studies in Epidemiology) flow chart of participant recruitment and study involvement considering exposure to the same VR environment in an observational design. VR: virtual reality.

A total of 100 volunteers (80 female participants and 20 male participants) participated in the study. Table 1 outlines demographic characteristics as well as scores of main study outcomes. The STROBE (Strengthening the Reporting of Observational Studies in Epidemiology [68]) flow chart in Figure 2 depicts the enrollment and study flow.

**Table 1.** Demographic characteristics and descriptive statistics of the full study sample (N=100).^a^

	Mean (SD)	Median (IQR)	Range
Age (y)	23.2 (1.70)	23.0 (22-24)	19-28
Education (y)	16.4 (1.09)	17.0 (16-17)	13-18
DASS-D^b^	9.4 (8.21)	6.0 (2-14)	0-30
DASS-A^c^	6.0 (6.22)	4.0 (0-10)	0-28
DASS-S^d^	12.7 (7.79)	12.0 (6-18)	0-36
CEI total^e^	31.1 (6.52)	31.5 (26-36)	13-45
CEI stretching^f^	17.1 (3.65)	17.0 (14.75-20)	6-24
CEI embracing^g^	14.0 (3.78)	14.0 (11-17)	6-21
SSQ^h^	30.2 (28.05)	22.4 (7.48-44.88)	0-119.7
Variety of objects	7.1 (5.78)	5.0 (3-9)	2-29
Time with objects (s)	60.3 (57.77)	34.9 (22.37-80.32)	3.2-257.2
Number of objects	12.6 (10.46)	9 (5-15.25)	2-61
Keyhole^i^ (s)	25.3 (43.58)	13.7 (8.34-22.24)	0-296.5
Time exploring^j^ (s)	1017.7 (383.5)	984.4 (728.4-1168.1)	380.9-2304.6
Number of gunshots^k^	4.0 (7.75)	0.0 (0-4.25)	0-37

a Male participants, n=20; female participants, n=80; participants currently taking antidepressant medication, n=1.

b DASS-D: Depression, Anxiety and Stress Scales-Depression.

c DASS-A: Depression and Stress Scales-Anxiety.

d DASS-S: Depression, Anxiety and Stress Scales-Stress.

e CEI total: Curiosity and Exploration Inventory-II total scale.

f CEI stretching: Curiosity and Exploration Inventory-II stretching subscale.

g CEI embracing: Curiosity and Exploration Inventory-II embracing subscale.

h SSQ: Simulator Sickness Questionnaire.

i Time spent looking through the keyhole.

j Time exploring: time spent in the virtual reality environment in addition to the cognitive tasks.

k Shot with gun, n=33; did not shoot with gun, n=67.

### Hypothesis 1

**Figure 3.**
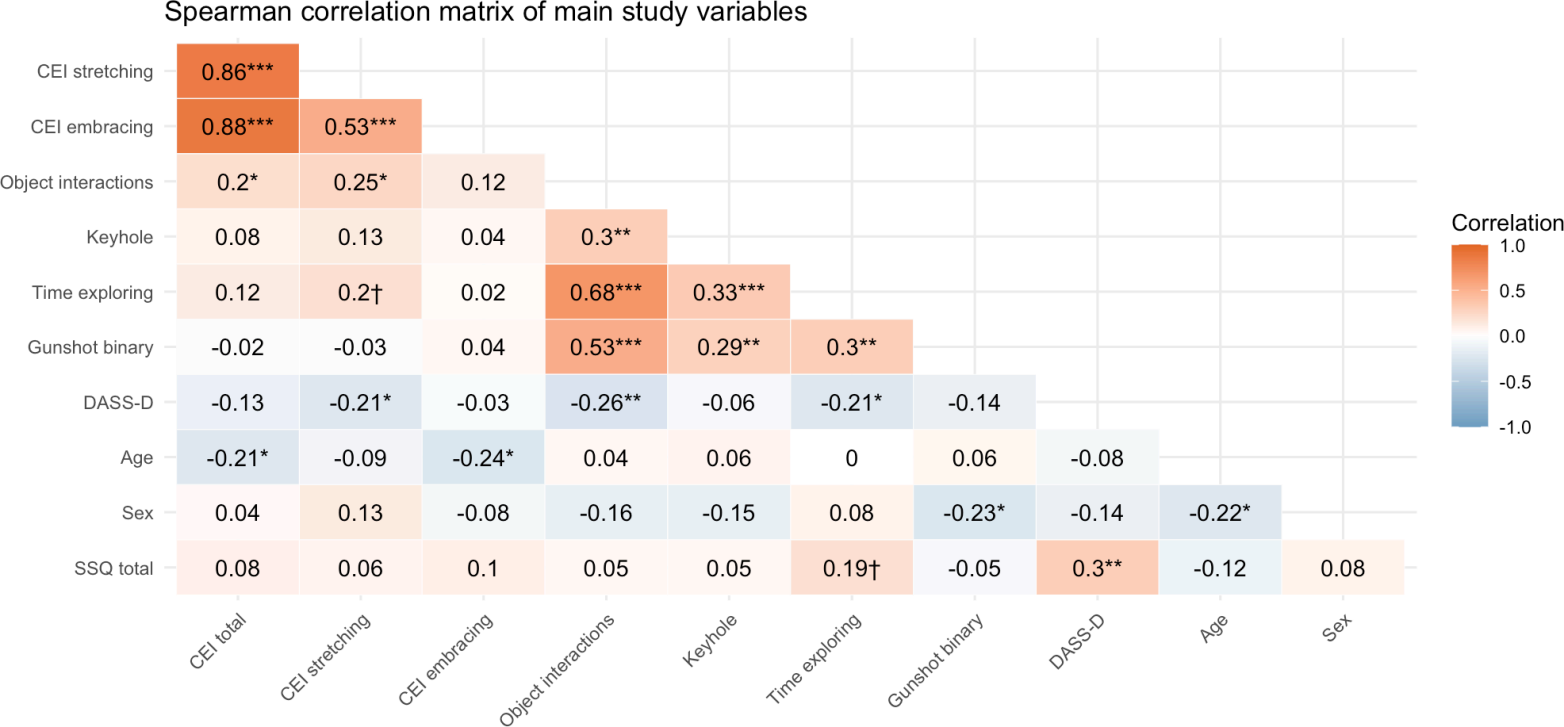
Spearman correlations among primary self-reported and behavioral study variables. Psychological self-report variables include DASS-D; CEI-II (total, stretching, and embracing subscales); and SSQ. Behavioral variables include object interactions, which represent a rank-based composite of object variety, time spent interacting, and number of objects combined; time exploring reflects exploration duration excluding required cognitive tasks; gunshot (binary) indicates whether participants discharged the laser gun; and keyhole represents time spent looking through the keyhole. Significance levels (based on unadjusted *P* values): ***P<.001, **P<.01, *P<.05, †*P*<.10. CEI-II: Curiosity and Exploration Inventory-II; DASS-D: Depression, Anxiety and Stress Scales-Depression; SSQ: Simulator Sickness Questionnaire.

Figure 3 shows the correlations among the VR curiosity variables and the CEI-II self-reported curiosity, as well as DS as assessed with DASS-D and included control variables. As stated, hypothesis 1 proposed that curiosity measured in VR would be positively associated to curiosity measured by the CEI-II. This was not supported, as only object interactions showed a significant correlation with the CEI-II total score (ρ=0.20, *P*=.04), which appeared to have mainly been driven by the correlation between object interactions and the Curiosity and Explorations Inventory (CEI)–stretching subscale (ρ=0.25, *P*=.01), as the association between CEI-embracing and object interactions was not significant (ρ=0.12, *P*>.10). However, after FDR adjustment, none of these associations remained statistically significant, warranting considerable uncertainty.

### Hypothesis 2

Hypothesis 2 stated that VR-curiosity would be inversely associated with DS. As seen in Table 2, when only the control variables were included in the base model, the SSQ was significantly associated with DS, explaining about 7% of the variance. Adding object interactions in model 1 doubled the explained variance to 14%, with object interactions (*Z*=−3.78, *P*<.001), the SSQ (*Z*=3.27, *P*<.001), and sex (*Z*=−2.0, *P*=.046) showing independent associations to DASS-D scores. In subsequent models, the additional VR curiosity indexes were added to model 1 in a stepwise manner and only kept if significant. However, as depicted in Table 2, neither time exploring, gunshot (binary), nor time spent looking through the keyhole were significantly associated with depression, and neither of the variables improved model fit. Correction for multiple testing did not meaningfully change effect estimations, as object interactions remained the sole significant predictor (*P*_adjusted_<0.005), providing partial support for hypothesis 2.

**Table 2.** Robust forward stepwise regression analysis of associations between virtual reality–curiosity variables (object interactions, time spent with exploration, whether the laser gun was fired, and time spent looking through a keyhole with ambiguous chatter) and depressive symptoms (DASS-D).

Model and variable	Estimate (SE)	*Z* value	*P* value
Base model^a^			
Intercept	11.87 (10.10)	1.18	.24
Sex	−2.85 (2.21)	−1.29	.20
Age	−0.20 (0.40)	−0.50	.62
SSQ^b^	0.12 (0.04)	3.13	.002
Model 1^c^			
Intercept	16.98 (10.05)	1.69	.09
Object interactions^d^	−0.09 (0.02)	−3.78	<.001
Sex	−3.91 (1.96)	−2.0	.046
Age	−0.18 (0.40)	−0.46	.65
SSQ	0.12 (0.03)	3.72	<.001
Model 2^e^			
Intercept	17.09 (10.32)	1.66	.10
Object interactions	−0.08 (0.04)	−2.03	.04
Time Exploring^f^	−0.001 (0.002)	−0.47	.64
Sex	−3.87 (1.95)	−1.98	.048
Age	−0.17 (0.40)	−0.42	.68
SSQ	0.12 (0.03)	3.76	<.001
Model 3^g^			
Intercept	17.54 (10.37)	1.69	.09
Object interactions	−0.08 (0.03)	−2.78	.005
Gunshot binary^h^	−1.16 (1.80)	−0.64	.52
Sex	−4.25 (2.27)	−1.88	.06
Age	−0.20 (0.41)	−0.48	.63
SSQ	0.12 (0.03)	3.91	<.001
Model 4^j^			
Intercept	16.56 (10.62)	1.56	.12
Object interactions	−0.09 (0.02)	−3.76	<.001
Keyhole^i^	−0.0002 (0.012)	−0.09	.98
Sex	−3.98 (2.03)	−1.96	.05
Age	−0.16 (0.43)	−0.37	.71
SSQ	0.12 (0.03)	3.68	<.001

a Robust *R*2=0.07.

b SSQ: Simulator Sickness Questionnaire.

c Robust *R*2=0.14.

d Object interactions: variety of objects, time spent with objects, and number of objects combined into 1 variable using rank-based methods.

e Robust *R*2=0.14.

f Time exploring: time spent exploring the environment excluding the required cognitive tasks.

g Robust *R*2=0.13.

h Gunshot binary: whether participants shot with the gun or not.

i Keyhole: time spent looking through the keyhole.

j Robust *R*2=0.14.

To further assess whether object interactions showed a unique association with DASS-D, and not its stress (DASS-S) and anxiety (DASS-A) components, sensitivity analyses were conducted where the same forward stepwise analyses were repeated for DASS-A and DASS-S as outcomes (outputs presented in Tables S1 and S2 in Multimedia Appendix 1). While the SSQ showed significant associations with both anxiety and stress symptoms (DASS-A, *Z*=3.69, *P*<.001; DASS-S, *Z*=3.03, *P*<.001), none of the VR curiosity indexes significantly related to DASS-A or DASS-S (Tables S1 and S2 in Multimedia Appendix 1), and neither did they improve model fit beyond the base model.

## Discussion

### Summary of Findings

This was, to our knowledge, the first study to explore the possibility of using VR in assessing exploratory behaviors as proxy measures of curiosity and their potential link to DS. More specifically, this study set out to test whether curiosity as assessed through four VR-based curiosity indices was associated with self-reported curiosity (hypothesis 1), and if these indices were inversely associated with DS (hypothesis 2). The first hypothesis was not supported, given that our results indicated that out of the 4 prespecified VR-based indices, only object interactions showed a weak association to the stretching aspect of curiosity, even though this did not retain significance after FDR corrections. Hypothesis 2, on the other hand, received partial support, given that object interactions were inversely associated with DS, controlling for sex, age, and cybersickness, robust to FDR corrections.

### Hypothesis 1

Our lack of support for the first hypothesis, while unexpected, indicated that our VR environment was not able to effectively capture curiosity as operationalized within the CEI-II [5]. This was specifically true for the CEI-embracing subscale that showed no association to the VR-based curiosity indices. In hindsight, these findings could be reflective of the fact that the embracing subscale taps into an *attitude toward life events* in general (eg, “I am the type of person who really enjoys the uncertainty of everyday life”) [5] and that this was something our VR environment—focusing on exploratory behaviors—clearly could not capture. Likewise, while a small positive correlation between object interactions and CEI-stretching was evident, this did not withstand correction for multiple testing, warranting uncertainty.

Nevertheless, with this uncertainty in mind, these findings could indicate a potential ability of object exploration within VR to capture a stretching aspect of curiosity, a construct that itself relates more to motivation to expand and explore [14]. These findings may suggest that the object interactions metric should be understood as potential behavioral correlates of curiosity-relevant exploratory motivation rather than a direct operationalization of curiosity per se. However, since we only validated our VR-based curiosity-metrics against 2 curiosity constructs—namely embracing and stretching, whether our VR scenario tapped into other curiosity dimensions, not captured by CEI-II [5], is difficult to infer. The limited convergence with self-reported curiosity is also consistent with literature, showing that trait curiosity and its behavioral manifestations are related but not interchangeable [3,27]. Nevertheless, given the abovementioned uncertainty of these exploratory findings, more research is needed to further expand on the possibilities of VR-based settings to assess a wider range of curiosity features.

### Hypothesis 2

Partial support for hypothesis 2 was somewhat consistent with the aforementioned findings, given that forward stepwise regression analysis indicated that out of the 4 curiosity indices, only object interactions significantly inversely related to DASS-D scores. In the final model, both object interactions and cybersickness demonstrated independent associations with DS. These findings were encouraging, indicating that the inverse relationship between object interactions and DS was not simply a reflection of greater cybersickness among individuals with higher levels of depressive symptoms. Importantly, the associations between object interactions and depressive symptoms remained statistically significant after FDR correction, indicating that the main findings were robust even after controlling for multiple testing.

These findings are in line with research on self-reported curiosity and affective symptoms, indicating associations with DS [14,15]. Our results on the sensitivity analysis are also consistent with studies, indicating that although associations between trait anxiety and aspects of curiosity have been reported [69,70], state anxiety has not been found to be associated with self-reported curiosity [71,72]—even though the relatively scarce number of studies on anxiety and curiosity should be noted. It is, however, also important to stress that while interactions with objects in our environment were associated with DASS-D scores, the other proposed VR indices (including overall time spent exploring the environment, whether participants decided to shoot with the gun, and time spent looking through the keyhole) were neither associated with self-reported curiosity nor depressive symptom levels. While we can only speculate about reasons for this, these results suggest that these measures were not appropriate as stand-alone VR-based curiosity metrics.

Moreover, while findings related to hypothesis 2 were encouraging, it is noteworthy that the overall explanatory power of the final model, which also included cybersickness symptoms, was relatively low (14%). Therefore, while the aim of this study was to hone in on VR-based curiosity and its association to DS, these findings underline the importance of including such metrics into multimodal evaluations incorporating physiological, eye-movement, as well as cognitive and emotional bias estimations to further enhance overall diagnostic precision, as demonstrated in [39].

### General Discussion and Future Directions

Taken together, this study is the first to demonstrate that spontaneous interactions with objects in a VR environment were associated with self-reported DS. Such results may open research avenues that could offer significant insights into advancing the field of curiosity research by demonstrating feasibility in assessments linking quantifiable, real-time behavioral measures to a psychological construct traditionally assessed via self-report [5,14,22]. Assessing exploration behaviors within a VR setting comes with several strengths, including its ability to offer stronger ecological validity that can enable more reliable insights into spontaneous exploration behaviors while avoiding subjective recall distortions. Moreover, as the VR scenario included the completion of cognitive tasks to move between rooms, participants were not aware that their exploration behaviors outside of these tasks were assessed, thus also avoiding issues relating to demand characteristics [25].

Given that a lower propensity to interact with objects was associated with higher levels of DS, but not anxiety or stress, this study also provides preliminary empirical support for the assertion that curiosity, operationalized through real-time exploratory behavior, is disrupted in depression. While these findings need to be built on, this may suggest that reduced engagement with novel stimuli or environments could serve as a behavioral marker of depression, distinct from generalized distress or hypervigilance seen in anxiety [73]. This study also adds complementary empirical insights to existing findings, suggesting that VR may be a potent tool for identifying differential cognitive changes in anxiety and depression [50], by adding knowledge on motivational and exploratory mechanisms related to DS. However, given this speculation, it is important to emphasize that participants in this study reported relatively low symptom levels on the DASS-21 scale. Therefore, whether our findings—not only regarding object interactions and DS but also the absence of associations to anxiety and stress—apply to more severe affective symptom profiles remains to be evaluated. Furthering research avenues where VR can help improve mental health assessment would also complement current progress seen within the use of VR for therapeutic and treatment purposes [40,74]. It is, however, important to highlight that user feedback regarding VR-based diagnostic solutions has indicated that such technologies should be used as a complement to traditional self-report measures and clinical interviews to improve assessment precision, as opposed to being used as stand-alone solutions [38].

While our findings have limited clinical applicability, our results could nevertheless offer etiological insights into depression—particularly its early stages and risk indicators. While the association between object interactions and self-reported curiosity was not generally supported, our results align with theories positing that depression involves a breakdown in motivated exploration and reward learning, where reduced object interactions may reflect a blunted response to potential environmental rewards or a deficit in goal-directed behavior, consistent with anhedonia and behavioral inhibition features of depression [75-77]. Moreover, given the nonclinical nature of our study, the substantial majority of our sample did not take antidepressant medication. This excludes the possibility that our findings were attributable to antidepressant medication side-effects that have been reported to reduce motivation [78]. However, future VR-based environments should further extend these findings through longitudinal assessments, to investigate whether a lower tendency toward exploration precedes the development of DS. Such findings could offer additional insights into relevant risk factors, supporting early detection and intervention, long identified as key to minimizing the negative impacts of depression [79-81].

### Limitations

Our study has several limitations that need to be considered. First, while we controlled for sex, age, and cybersickness, a key limitation is that we did not take prior VR and gaming experience into account. As prior VR experience may influence the propensity to which individuals engage with various elements within VR environments, future studies should consider this important variable. This is not only essential to improve study rigor but could also give insight into potential barriers toward patients using VR for mental health purposes—a currently understudied area [40].

Second, it is important to consider that while our VR environment aimed to offer an ecologically valid assessment of exploratory behaviors, the challenges inherent in the scenario (consisting of needing to complete a set of cognitive tasks), while not itself related to exploration, may nevertheless have artificially enhanced engagement and motivation beyond everyday life [82].

Third, some issues regarding the study sample need to be highlighted, as it was composed of highly educated young adults, the majority being female participants, which limits generalizability. For instance, exploratory tendencies have been shown to decline with age, and age-related reductions on constructs related to curiosity have also been reported [83,84]. Higher education is associated with openness or intellect, conceptualized as cognitive exploration [85], which overlaps with trait curiosity as measured with the CEI-II [5]. Accordingly, generalization to older, less educated samples is limited. Furthermore, although overall gender differences in curiosity are generally small, domain-specific patterns have been reported, with women tending to show greater interpersonal curiosity and men greater object-oriented or investigative interests [86]. Moreover, given we did not include a clinical sample with a confirmed major depressive disorder diagnosis, nor looked at symptom severity thresholds, this represents a limitation in terms of elucidating clinically meaningful applications of our findings. Additionally, it is important to highlight that our sample was not only limited in terms of an absence of a confirmed clinical diagnosis but also by the fact that the majority reported symptoms in the “normal” range. Therefore, further studies are needed to evaluate whether our findings apply to clinical samples—particularly individuals with more severe symptom profiles. Conversely, our finding that interactive behavior was inversely linked to DS, even among individuals with a relatively low-moderate symptom profile, was encouraging, as it may suggest that studying curiosity behaviors in VR is an important avenue for improving methods to strengthen early detection of at-risk individuals.

Fourth, while we included several proposed curiosity metrics, future studies should consider involving more intricate and multimodal ways of assessing curiosity that may also tap into other constructs, such as social curiosity or deprivation sensitivity [14].

Fifth, it should be noted that using the SSQ, a scale originally designed for flight simulators, within VR has received criticism [87]. For instance, its psychometric properties within VR have been questioned [88-90], as has its 0-baseline symptom assumption [91] and limited differentiation from affective somatic symptoms [38]. As no baseline assessment was conducted in this study (consistent with the original validation of the SSQ [61]), symptom severity cannot be unequivocally attributed to VR exposure alone. As discussed in greater detail elsewhere [38], baseline-adjusted designs with VR-specific instruments that better differentiate cybersickness from somatic symptoms of affective disorders may improve construct validity in future research and are necessary to clarify the relationship between cybersickness, curiosity, and DS (eg, [89]).

Finally, while the current VR environment lent itself well to a cross-sectional examination of spontaneous exploratory behaviors, future studies should look to develop VR environments that enable longitudinal assessments of curiosity, as this would allow researchers to track dynamic changes in exploratory behavior over time, which could provide deeper insights into the stability and evolution of curiosity and its influence on mental health outcomes. In addition to longitudinal extensions, VR environments offer opportunities to broaden the dimensional scope of curiosity assessment. Beyond object-interaction indices, immersive platforms allow for the integration of fine-grained spatial trajectory metrics (eg, center-periphery preferences, movement dynamics, and revisit patterns), analogous to human adaptations of open-field paradigms. Such spatial measures have been shown to be associated with psychiatric symptom profiles in both physical and virtual paradigms [28,29] and may complement interaction-based metrics in future multidimensional models of exploratory behavior in VR.

### Conclusion

This study provides preliminary insights into the potential of VR to offer an ecologically valid estimation of spontaneous exploration behaviors, as they occur in real time. These findings should be built upon, as they can be a promising avenue for furthering insights into the etiology of depressive symptomatology. In particular, future longitudinal studies with more sophisticated, ideally multimodal curiosity-based metrics are needed to better entangle the intricate relationship between curiosity-driven motivations and the development of DS.

## Supplementary material

10.2196/80120 Multimedia Appendix 1 Tutorial script, Spearman correlation matrix among VR-curiosuty variables, sensitivity analysis output.

## Abbreviations

CEI: Curiosity and Explorations Inventory
CEI-II: Curiosity and Exploration Inventory-II
DASS-21: Depression Anxiety and Stress Scale-21 items
DASS-A: Depression and Stress Scales-Anxiety
DASS-D: Depression, Anxiety and Stress Scales-Depression
DASS-S: Depression and Stress Scales-Stress
DS: depressive symptoms
FDR: false discovery rate
HC: healthy control
PHQ-9: Patient Health Questionnaire-9
SSQ: Simulator Sickness Questionnaire
STROBE: Strengthening the Reporting of Observational Studies in Epidemiology
VR: virtual reality
