# Supplementary material for: Curiosity in a Novel Virtual Reality Scenario and Its Association With Symptoms of Depression: Observational Pilot Investigation
Source: JMIR Form Res. 2026 May 4;10:e80120. doi: 10.2196/80120 (PMC13138711; doi:10.2196/80120)
Supplement: Multimedia Appendix 1 [file formative-v10-e80120-s001.docx]

Supplementary materials

VR-Tutorial script

**Welcome**

You unexpectedly wake up in an unknown hallway. You have no recollection of why and how you ended up there. It feels like a typical office space.

There isn’t much else but a single door in front of you. It looks like there is some kind of device that can be used to unlock the door.

**Tutorial**

The objective of this tutorial is to get familiarized with the controller and the interactions that will take place during the experience.

Look at the remote, the right button will be highlighted in each step.

To interact with the panels, you will use a beam. If you want to select something you just have to aim and then press the Trigger button (see the remote).

TASK 1: aim at the upper right cross and press the button.

Move around the scenario

Next, we will learn how to move around the instance.

A lightning rod will appear indicating the destination, release to teleport

TASK: Teleport to the back table

Elements Interaction

During the experience there are many elements to interact with. Try to pick up the objects in front of you (look at the controller)

TASK: Pick up the objects.

Tutorial ended

Once you approach the door, there will be a task for you to solve.

The door will be unlocked once you complete the task.

The room behind the door will contain further clues as to how you can progress and eventually get out of this place. To unlock the other doors, you will need to complete short tasks (one task for each door).

Take your time and look around, you might find some things to explore. The door that leads to the outside will be marked with a big EXIT sign and will unlock only after all tasks are completed.

Figure S1.

Spearman’s correlation matrix among VR curiosity variables


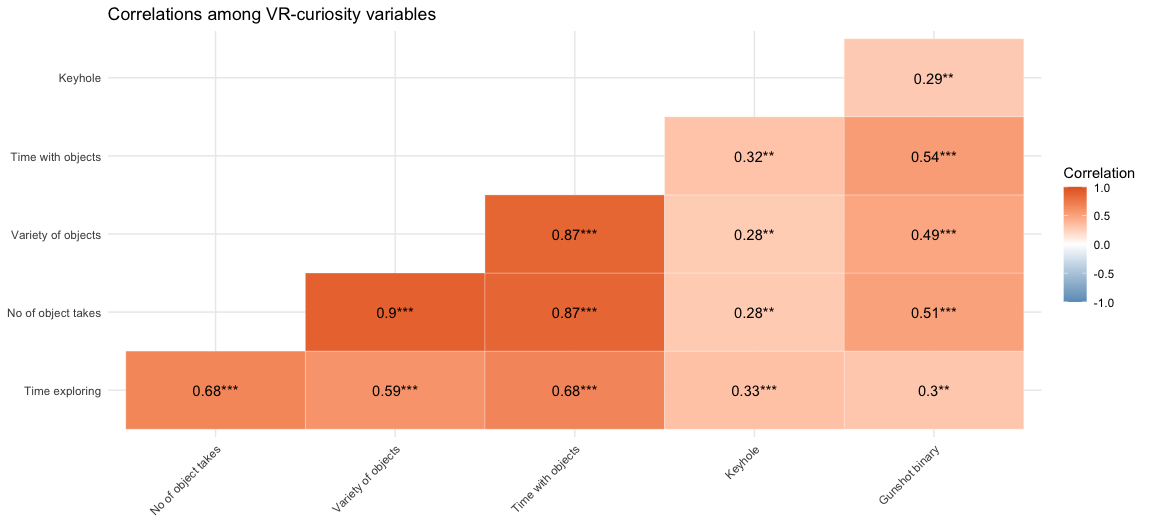


Notes: *** *P* < .001, ** *P* < .01*, * P* < .05*.* Variety of objects = Variety of objects interacted with; Time with objects = time spent interacting with objects; No of object takes = number of times objects were picked up; Time exploring = time spent exploring the environment excluding the required cognitive tasks; Keyhole = time spent looking through the keyhole; Gunshot binary = whether participants shot with the gun or not.

Table S1. Robust forward stepwise regression analysis of VR-curiosity variables predicting anxiety (DASS-A).

| **Base Model** | **Estimate** | **Std. Error** | **z-value** | ***P*** |
| --- | --- | --- | --- | --- |
| Intercept | 7.64 | 9.86 | 0.78 | .438 |
| Sex | − 2.54 | 1.68 | −1.51 | .131 |
| Age | − 0.12 | 0.39 | −0.30 | .77 |
| SSQ^a^ | 0.074 | 0.02 | 3.69 | <.001 |
| Robust R^2^ = 0.08 | | | | |
| **Model 1** | **Estimate** | **Std. Error** | **z-value** | ***P*** |
| Intercept | 8.34 | 9.33 | 0.89 | .372 |
| Object interactions^b^ | − 0.007 | 0.02 | − 0.34 | .732 |
| Sex | − 2.58 | 1.59 | − 1.63 | .103 |
| Age | − 0.13 | 0.38 | − 0.34 | .737 |
| SSQ^a^ | 0.07 | 0.02 | 3.87 | <.001 |
| Robust R^2^ = 0.07 | | | | |
| **Model 2** | **Estimate** | **Std. Error** | **z-value** | ***P*** |
| Intercept | 7.43 | 9.87 | 0.75 | .452 |
| Time Exploring^c^ | 0.0009 | 0.002 | 0.545 | .586 |
| Sex | −2.55 | 1.73 | −1.47 | .140 |
| Age | − 0.14 | 0.40 | −0.36 | .72 |
| SSQ^a^ | 0.07 | 0.02 | 3.82 | <.001 |
| Robust R^2^ = 0.08 | | | | |
| **Model 3** | **Estimate** | **Std. Error** | **z-value** | ***P*** |
| Intercept | 7.48 | 9.83 | 0.76 | .45 |
| Gunshot binary^d^ | 0.51 | 1.12 | 0.45 | .651 |
| Sex | − 2.46 | 1.69 | − 1.45 | .148 |
| Age | − 0.12 | 0.39 | − 0.31 | .760 |
| SSQ^a^ | 0.07 | 0.02 | 3.73 | <.001 |
| Robust R^2^ = 0.08 | | | | |
| **Model 4** | **Estimate** | **Std. Error** | **z-value** | ***P*** |
| Intercept | 9.25 | 9.50 | 0.97 | .33 |
| Keyhole^e^ | 0.001 | 0.009 | − 0.12 | .908 |
| Sex | − 2.35 | 1.62 | − 1.45 | .15 |
| Age | − 0.19 | 0.39 | − 0.49 | .624 |
| SSQ^a^ | 0.07 | 0.02 | 4.00 | <.001 |
| Robust R^2^ = 0.07 | | | | |

Notes: ^a^SSQ = Simulator Sickness Questionnaire; ^b^Object interactions = Variety of objects, time spent with objects and number of objects combined into one variable using rank-based methods; ^c^Time exploring = time spent exploring the environment excluding the required cognitive tasks; ^d^Gunshot binary = whether participants shot with the gun or not; ^e^Keyhole = time spent looking through the keyhole.

Table S2. Robust forward stepwise regression analysis of VR-curiosity variables predicting stress (DASS-S).

| **Base Model** | **Estimate** | **Std. Error** | **z-value** | ***P*** |
| --- | --- | --- | --- | --- |
| Intercept | 23.87 | 12.42 | 1.92 | .055 |
| Sex | − 1.40 | 1.91 | − 0.73 | .465 |
| Age | − 0.56 | 0.52 | − 1.08 | .282 |
| SSQ^a^ | 0.08 | 0.03 | 3.03 | .002 |
| Robust R^2^ = 0.07 | | | | |
| **Model 1** | **Estimate** | **Std. Error** | **z-value** | ***P*** |
| Intercept | 25.22 | 13.06 | 1.93 | .054 |
| Object interactions^b^ | − 0.03 | 0.03 | − 0.92 | .360 |
| Sex | − 1.74 | 1.98 | − 0.88 | .381 |
| Age | − 0.53 | 0.55 | − 0.97 | .334 |
| SSQ^a^ | 0.08 | 0.03 | 2.96 | .003 |
| Robust R^2^ = 0.08 | | | | |
| **Model 2** | **Estimate** | **Std. Error** | **z-value** | ***P*** |
| Intercept | 24.74 | 12.78 | 1.94 | .053 |
| Time Exploring^c^ | − 0.001 | 0.002 | − 0.50 | .619 |
| Sex | − 1.39 | 1.95 | − 0.71 | .477 |
| Age | − 0.55 | 0.55 | − 0.99 | .324 |
| SSQ^a^ | 0.08 | 0.03 | 2.98 | .003 |
| Robust R^2^ = 0.07 | | | | |
| **Model 3** | **Estimate** | **Std. Error** | **z-value** | ***P*** |
| Intercept | 24.08 | 12.81 | 1.88 | .06 |
| Gunshot binary^d^ | − 0.76 | 1.78 | − 0.43 | .667 |
| Sex | 1.64 | 2.08 | − 0.79 | .431 |
| Age | − 0.54 | 0.53 | − 1.02 | .308 |
| SSQ^a^ | 0.08 | 0.03 | 2.98 | .003 |
| Robust R^2^ = 0.07 | | | | |
| **Model 4** | **Estimate** | **Std. Error** | **z-value** | ***P*** |
| Intercept | 22.61 | 13.54 | 1.67 | .095 |
| Keyhole^e^ | − 0.002 | 0.012 | − 1.20 | .844 |
| Sex | − 1.57 | 1.99 | − 0.79 | .428 |
| Age | − 0.49 | 0.57 | − 0.85 | .394 |
| SSQ^a^ | 0.08 | 0.03 | 2.81 | .005 |
| Robust R^2^ = 0.06 | | | | |

Notes: ^a^SSQ = Simulator Sickness Questionnaire; ^b^Object interactions = Variety of objects, time spent with objects and number of objects combined into one variable using rank-based methods; ^c^Time exploring = time spent exploring the environment excluding the required cognitive tasks; ^d^Gunshot binary = whether participants shot with the gun or not; ^e^Keyhole = time spent looking through the keyhole.
